# *De ja vu?* Post-COVID-19 Surge in Respiratory Illnesses Among Children in China Emphasizes Need for Proactive Surveillance, Openness, Early Detection and Reporting of Causative Pathogen(s), and Their AMR Status

**DOI:** 10.1007/s44197-023-00177-2

**Published:** 2023-12-11

**Authors:** David S. Hui, Alimuddin Zumla, Ziad A. Memish

**Affiliations:** 1 Department of Medicine and Therapeutics and S. H. Ho Research Center for Infectious Diseases, The Chinese University of Hong Kong, Shatin, New Territories, Hong Kong, China; 2 Department of Infection, Division of Infection and Immunity, Centre for Clinical Microbiology, University College London, and NIHR Biomedical Research Centre, University College London Hospitals, London, UK; 3 Research and Innovation Center, King Saud Medical City, Ministry of Health and College of Medicine, Al Faisal University, Riyadh, Saudi Arabia; 4 Hubert Department of Global Health, Rollins School of Public Health, Emory, University, Atlanta, USA; 5 Kyung Hee University, Seoul, South Korea

Recent alarming media reports of surges in respiratory illnesses among children in Northern China showing crowded hospital hallways with children receiving intravenous infusion raised global concern and *de ja vu* of a new pathogen, as was the case with in Dec 2019 [1]. At a press conference on 13 November 2023, the Chinese National Health Commission reported an increase in incidence of respiratory diseases in China, attributing this upsurge to lifting of COVID-19 restrictions and subsequent increased circulation of known respiratory tract pathogens, such as influenza, respiratory syncytial virus (RSV), *Mycoplasma pneumoniae*, and SARS-CoV-2 [2] as seen in USA and other countries. On 20 November 2023, ProMed, a publicly available online surveillance program, reported that hospitals in Beijing, Liaoning, and other cities were overwhelmed with schools and classes on the verge of suspension [3]. Following a request by the WHO on 22 Nov 2023, the Chinese Health Authorities provided further information on 23 Nov 2023, stating an increase in out-patient consultations and hospital admissions for children with *M. pneumoniae* had occurred since May 2023 followed by a downward trend after reaching the peak in September, while surges in influenza A/H3N2, adenovirus, and RSV have occurred since Oct 2023 [4]. *M. pneumoniae* had been detected mainly among those aged 5–14 yrs, while the rest of the population was affected by a variety of respiratory viruses [5]. Reassuringly, no new pathogen was detected and there was no increase in resistance of influenza viruses to oseltamivir [6]. However, the global alarm bells in lieu of the ongoing outbreak in China raise several important issues for ongoing global public health dialog on proactive surveillance, openness, efficient communication, early detection and reporting of causative pathogen(s), their AMR status for effective management, and control of respiratory tract infections (RTIs).

While China was well known for its strict implementation of dynamic zero policy [7], with rapid lockdown of cities involved during the COVID-19 pandemic from 2020 to Dec 2022, COVID-19 mitigation measures were lifted abruptly. This, together with lack of exposure to a variety of common respiratory pathogens and immune escape during 3 years of restrictions when the COVID-19 pandemic was a public health emergency of international concern is not surprising to see upsurges of influenza and other common respiratory pathogens [8]. Since mid-Oct 2023, China has enhanced the surveillance systems for respiratory infections due to respiratory viruses and bacteria including *M. pneumoniae* [4]. It is important to note that resistance of *M. pneumoniae* to macrolide has been an-going problem in Beijing, likely due to extensive use of macrolide in recent decades. A surveillance study showed that macrolide resistance rates of *M. pneumoniae* in the Beijing population were as high as 68.9%, 90.0%, 98.4%, 95.4%, and 97.0% in the years 2008 to 2012, respectively. Common macrolide-resistant mobile genetic elements were not detected with any isolate. These macrolide-resistant isolates came from multiple clones rather than the same clone and a large aggregation of a particular clone was not detected in a specific period [9]. In a study investigating an outbreak of *M. pneumoniae* in a primary school in Beijing in 2018, 25 out of 55 cases required hospitalization while 72% (18/25) of inpatients had radiographic findings consistent with pneumonia, and some cases were hospitalized for up to 4 weeks. Pathogen detection results indicated that *M. pneumoniae* P1 type 1 was the causative agent in this outbreak, and the strain harbored one-point mutation of A to G at position 2063 [10]. Resistance to macrolides in *M. pneumoniae* has been emerging worldwide since the mid-2000s. Over the last 20 years, the prevalence of macrolide-resistant *M. pneumoniae* has remained high in China with a significant increasing trend in South Korea, from 4 to 78% [11]. Resistance rates up to 90% have been reported in children with *M. pneumoniae* infection increasing during COVID-19 pandemic [12]. In the United States and Europe, macrolide resistance in *M. pneumoniae* rates may be up to 10% with regional variability.

Surges of childhood pneumonia post-COVID-19 have also been reported recently in the Netherlands in parallel with the outbreak in China [13]. It is important to enhance disease surveillance in healthcare facilities and community settings, in addition to strengthening the capacity of the health system to manage patients. To reduce the risk of spreading respiratory illness, the WHO has recommended that people in the affected areas stay up to date with immunization especially with influenza and COVID-19 vaccines, maintain social distancing from ill people, stay home when sick, seek medical attention as needed, wear masks as appropriate, and pay attention to hand hygiene regularly [4].

When overwhelming numbers of cases with RTIs occur widespread empiric overuse of antibiotics inevitably follows. Antimicrobial stewardship and proactive surveillance remain important strategies in controlling the global problem of antimicrobial resistance which is accelerated by the misuse and overuse of antibiotics during epidemics of respiratory infections [14]. With advancing developments in accurate multiplex diagnostic screening for multiple pathogens simultaneously from a single clinical samples, further investments into development of more accurate, affordable, easy to use point-of-care diagnostic tests are required. This will enable rapid identification of pathogen, its AMR status, and lead to early reporting to WHO, preventing the *de ja vu* of global hysteria when outbreaks occur.

## Abbreviations

COVID-19: Coronavirus disease 2019
SARS-CoV-2: Severe acute respiratory syndrome-coronavirus-2
AMR: Antimicrobial resistance
WHO: World Health Organization
RTIs: Respiratory tract infections
